# Beta Trace Protein does not outperform Creatinine and Cystatin C in estimating Glomerular Filtration Rate in Older Adults

**DOI:** 10.1038/s41598-017-12645-4

**Published:** 2017-10-04

**Authors:** Natalie Ebert, Camilla Koep, Kristin Schwarz, Peter Martus, Nina Mielke, Jan Bartel, Martin Kuhlmann, Jens Gaedeke, Markus Toelle, Markus van der Giet, Mirjam Schuchardt, Elke Schaeffner

**Affiliations:** 1 Institute of Public Health, Charité University Medicine, Berlin, Germany; 2 Institute of Clinical Epidemiology and Medical Biostatistics, Eberhard Karls University, Tübingen, Germany; 3 Limbach Laboratory, Heidelberg, Germany; 4 Department of Nephrology, Vivantes Klinikum im Friedrichshain, Berlin, Germany; 5 Division of Nephrology, Charité University Medicine, Campus Mitte, Berlin, Germany; 6 Division of Nephrology, Charité University Medicine Campus Benjamin Franklin, Berlin, Germany

**Keywords:** Diagnostic markers, Kidney diseases

## Abstract

Despite intense research the optimal endogenous biomarker for glomerular filtration rate (GFR) estimation has not been identified yet. We analyzed if ß-trace protein (BTP) improved GFR estimation in elderly. 566 participants aged 70+ from the population-based Berlin Initiative Study were included in a cross-sectional validation study. BTP, standardized creatinine and cystatin C were measured in participants with iohexol clearance measurement as gold standard method for measured GFR (mGFR). In a double logarithmic linear model prediction of mGFR by BTP was assessed. Analyses with BTP only and combined with creatinine and cystatin C were performed. Additionally, performance of GFR estimating equations was compared to mGFR. We found that the combination of all three biomarkers showed the best prediction of mGFR (r^2^ = 0.83), whereat the combination of creatinine and cystatin C provided only minimally diverging results (r^2^ = 0.82). Single usage of BTP showed worst prediction (r^2^ = 0.67) within models with only one biomarker. Subgroup analyses (arterial hypertension, diabetes, body mass index ≤23 and >30) demonstrated a slight additional benefit of including BTP into the prediction model for diabetic, hypertensive and lean patients. Among BTP-containing GFR equations the Inker BTP-based equation showed superior performance. Especially the use of cystatin C renders the addition of BTP unnecessary.

## Introduction

Glomerular filtration rate (GFR) is considered the best indicator of kidney function^1^. It can either be estimated (eGFR) using mathematical equations based on endogenous biomarkers or it can be measured (mGFR) using invasive methods based on the injection of exogenous markers. At present, in clinical routine the majority of health care providers use serum creatinine levels to calculate eGFR. Recently, it has been shown that, especially in older adults and children, adding cystatin C as another renal biomarker to creatinine level for assessing GFR increases the accuracy of eGFR as compared to the use of single-biomarker eGFR^2–4^. A rising number of novel biomarkers have emerged as alternative to creatinine and cystatin C^5,6^, one of them ß-trace protein (BTP), a heterogeneous low molecular weight glycoprotein produced in the central nervous system^7^. Concentrations in serum however are found to be lower^8^. Since BTP’s discovery about 20 years ago research has focused on its possible ability to shed additional light on assessing kidney function beyond serum creatinine (Scr) and serum cystatin C (SCys)^9^. With regard to its renal metabolism, BTP has been shown to be freely filtered through the glomerular basement membrane with little if any tubular reabsorption or non-renal elimination^7^. Also, ß2-microglobulin (B2M), another low molecular weight protein, has been found to be highly correlated with measured GFR (mGFR) and like BTP, is less affected by age and sex^10,11^. Other research groups have investigated the approach of “biomarker-panels” using several, still unspecified, markers simultaneously, although these markers are not yet established for standard laboratory analysis^12^.

For the estimation of GFR it is important to know whether the renal marker in use performs equally well in different patient populations, such as children, older adults, individuals with liver cirrhosis or muscle wasting, and transplanted patients. Factors that are known to influence a renal biomarker’s potential to correctly estimate GFR are called the non-GFR determinants (e.g. muscle mass, age, gender, and diet). Although creatinine-based GFR equations give reliable estimates in adult patients with reduced kidney function, it is known that both, for children and for older adults, continuous change in muscle mass leads to unreliable Scr levels for GFR estimation^13,14^. In this respect, BTP has been shown to have potentially superior properties for assessing kidney function, especially in renal transplant recipients^15,16^. Whether BTP may increase the accuracy of GFR estimates when combined with existing markers applied to older adults is still unknown.

In the present study we hypothesized that in old age adding BTP to single or the combination of current biomarkers improved GFR estimation compared to using Scr or Scys alone. In a double logarithmic linear model prediction of mGFR by BTP only and combined with creatinine and cystatin C was assessed. Additionally, we evaluated current BTP-containing eGFR equations against measured GFR and in comparison to combined creatinine/cystatin C-based equations in a population of older adults.

## Results

**Figure 1:**
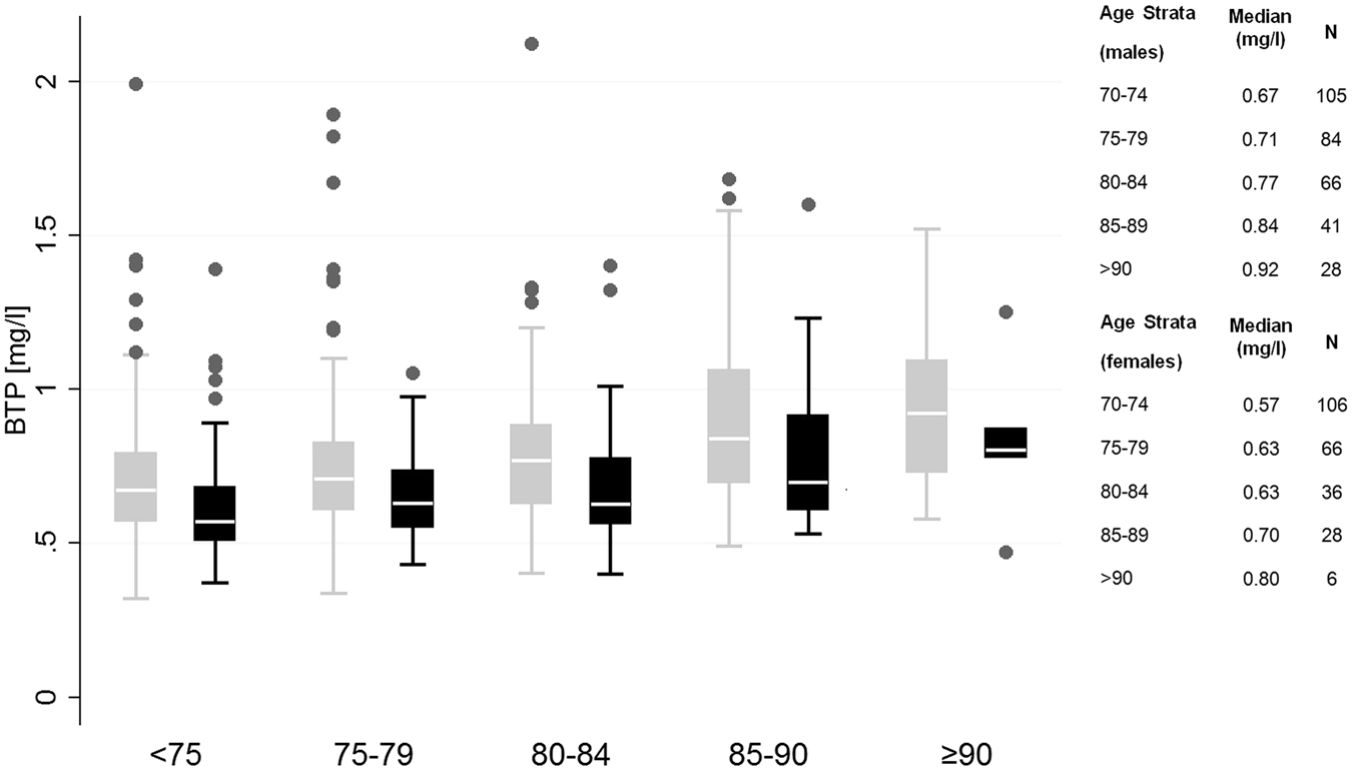
Median BTP concentration by age and gender in the BIS iohexol population (n = 566). Age strata 70–75 years up to ≥90 years, males are in grey and females in black. Boxes indicate medians (line inside box), quartiles (upper and lower margins of box). Antennae are defined by the rule upper-lower box margin ± 1.5x interquartile range. Circles indicate outliers. Exact median BTP concentrations per age stratum for males and females are given in mg/l next to the boxplots.

In the BIS iohexol population with measured BTP (Table 1), mean age (±SD) was 78.5 (6.2) years, 43% were female, one quarter had diabetes, more than three quarters had hypertension, and nearly one third was overweight (body mass index ≥30 kg/m^2^). Median serum concentration of BTP in the total cohort was 0.68 mg/l with 0.73 mg/l in males and 0.62 mg/l in females. For creatinine it was 0.91 mg/dl with 1.02 in males and 0.79 in females and for cystatin C it was 1.05 mg/dl with 1.1 mg/dl in males and 0.99 mg/dl in females. Figure 1 shows an increase of BTP concentration by age category: in 70–75 year old patients mean BTP was 0.57 mg/L in females and 0.67 mg/dl in males as compared to age >90 years with mean BTP of 0.80 mg/L in females and 0.92 mg/L in males, corresponding to a 14% increase of BTP concentration between the lowest and the highest age stratum for male and females.

**Table 1:** Main Characteristics of the Iohexol Population with BTP.

**Characteristics**	**Total Sample**
Participants, n	566
Mean age, y (SD)	78.5 (±6.2)
Female, n (%)	242 (42.8)
Diabetes mellitus, n (%)^1^	136 (24.0)
Arterial hypertension, n (%)^2^	435 (76.9)
Body mass index, n (%)	
<25 kg/m^2^	150 (26.5)
25–29.9 kg/m^2^	258 (45.6)
≥30 kg/m^2^	158 (27.9)
Mean BSA (range), m^2^	1.85 (1.4–2.4)
Mean serum BTP (range), mg/L	0.75 (0.32–4.25)
Mean serum creatinine level (range) µmol/L mg/dl	87.9 (40.7–421.7) 0.99 (0.46–4.77)
Mean serum cystatin C level (range) mg/L	1.14 (0.61–4.40)
Mean hemoglobin (range), g/dl^3^	13.6 (9.5–19.1)
Mean albumin (range), g/L^4^	40.3 (30.8–51.9)
Mean c-reactive protein (range), mg/L^5^	3.40 (0.23–44.21)
Mean mGFR (range) ml/min/1.73 m^2^	60.4 (15.5–116.7)
Mean eGFR_Pöge_BTP_ (range) ml/min/1.73 m^2^	63.9 (15.0–116.5)
Mean eGFR_Pöge_BTP/Crea_ (range) ml/min/1.73 m^2^	62.8 (13.4–111.8)
Mean eGFR_Inker_BTP_ ml/min/1.73 m^2^	58.4 (15.5–105.0)
Mean eGFR_White_BTP/Crea_ ml/min/1.73 m^2^	86.24 (14.2–163.0)

mGFR = measured glomerular filtration rate; ^1^Diabetes was defined as either HbA1c >6.5%, and/or prescription of antidiabetic medication. ^2^Hypertension was defined as prescription of antihypertensive medication. ^3^To convert cystatin C from mg/L to nmol/L, multiply by 74.9; ^4^To convert hemoglobin from mmol/L to g/dL, divide by 0.621; ^5^To convert albumin from g/L to g/dL, multiply by 0.1; ^6^To convert C - reactive protein from mg/L to mg/dL, multiply by 0.1.

### Performance of BTP alone and in combination with creatinine and cystatin C and compared to mGFR

All filtration markers were negatively correlated with measured GFR (mGFR) (Supplement Table 1) ranging from −0.78 (creatinine) to −0.78 (BTP) and −0.87 (cystatin C). Comparison of renal markers showed the highest correlation between BTP and cystatin C (0.83) followed by creatinine and cystatin C (0.82) and the lowest between BTP and creatinine (0.77). In the regression model adjusted for age, gender and body mass index (BMI) (Table 2) the single usage of BTP showed the worst prediction (r^2^ = 0.632) within the models with only one biomarker. The age, gender, and BMI-adjusted combination of BTP, creatinine and cystatin C showed the best prediction of mGFR (r^2^ = 0.833), whereat the adjusted combination of creatinine and cystatin C provided only minimally diverging results (r^2^ = 0.828). Additionally, the combination of creatinine and BTP (r^2^ = 0.789) did not outperform the combination of creatinine and cystatin C.

**Table 2:** Seventeen Regression Models including either Single Renal Biomarker or their Combination in Individuals aged 70 years and above (n = 566).

**Regression model**	**Model Fit**	
	**corrected R** ^**2**^ **(95% CI)**	**RMSE (95% CI)**
BTP	0.632 (0.565–0.698)	0.184 (0.169–0.198)
BTP, age, gender	0.671 (0.61–0.730)	0.174 (0.159–0.188)
BTP, age, gender, BMI	0.670 (0.608–0.732)	0.174 (0.161–0.187)
CysC	0.759 (0.718–0.799)	0.149 (0.138–0.159)
CysC, age gender	0.780 (0.746–0.815)	0.142 (0.132–0.151)
CysC, age gender, BMI	0.786 (0.751–0.821)	0.140 (0.131–0.149)
CysC + BTP, age, gender BMI	0.802 (0.770–0.835)	0.135 (0.126–0.143)
Crea	0.609 (0.547–0.670)	0.189 (0.175–0.203)
Crea, age, gender	0.740 (0.695–0.784)	0.154 (0.142–0.167)
Crea, age, gender, BMI	0.744 (0.694–0.794)	0.153 (0.141–0.165)
Crea + BTP, age, gender BMI	0.789 (0.751–0.827)	0.139 (0.129–0.149)
Crea + CysC,	0.772 (0.736–0.809)	0.144 (0.135–0.153)
Crea + CysC, age, gender	0.821 (0.792–0.850)	0.128 (0.118–0.138)
Crea + CysC, age, gender, BMI	0.828 (0.797–0.858)	0.126 (0.117–0.135)
Crea + CysC + BTP	0.782 (0.747–0.816)	0.141 (0.133–0.150)
Crea + CysC + BTP, age, gender	0.828 (0.799–0.859)	0.125 (0.117–0.134)
Crea + CysC + BTP, age, gender BMI	0.833 (0.804–0.862)	0.124 (0.115–0.132)

BMI = body mass index, BTP = beta trace protein; RMSE = Root Residual Mean Square Error. Confidence limits for R^2^ values and RMSE were calculated via bootstrap resampling.

### Validation of BTP-based eGFR equations

**Figure 2:**
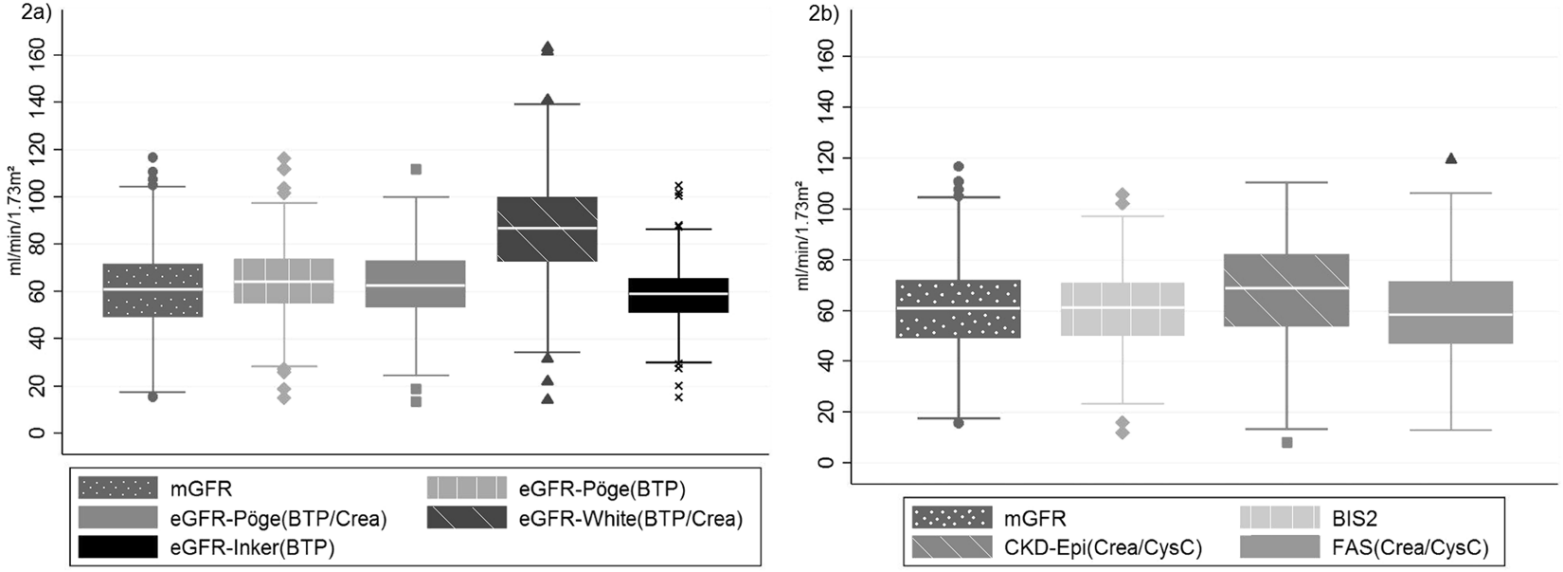
(**a**) Comparison of mGFR with the four BTP-based GFR equations. (**b**) Comparison of mGFR with combined creatinine/cystatin C GFR equations applicable in older adults. (**a**,**b**) Boxes indicate medians (line inside box), quartiles (upper and lower margins of box). Antennae are defined by the rule upper-lower box margin ± 1.5x interquartile range. Circles, squares and triangles indicate outliers. For estimating equations, refer to material section. mGFR = measured glomerular filtration rate; eGFR = estimated glomerular filtration rate; BIS = Berlin Initiative Study; CKD-Epi = Chronic Kidney Disease Epidemiology Collaboration, FAS = Full Age Spectrum.

Mean mGFR (range) was 60.4 (16–117) ml/min/1.73 m^2^ and compared to the estimated values using the equations described in the methods section we found rather heterogeneous results: the highest mean estimated GFR (eGFR) (range) of 86 (14–163) ml/min/1.73 m^2^ was found with the White_(BTP/Crea)_ equation compared to less biased BTP-based eGFR results of 58 (15–105), 63 (13–112), and 64 (15–117) for the Inker_(BTP)_, the Pöge_(BTP/Crea)_ and the Pöge_(BTP)_ equations, respectively. Figure 2a shows the boxplot of mGFR and eGFR, according to all four BTP-based equations. Compared to the median mGFR value of 61 ml/min/1.73 m^2^ the Inker_(BTP)_ equation showed the closest estimate with the smallest interquartile range. Both equations by Pöge slightly overestimated median mGFR. The estimate of the White_(BTP/Crea)_ equation largely overestimated mGFR and showed the widest interquartile range. Figure 2b shows the boxplot of mGFR and eGFR, according to the three creatinine/cystatin C-based equations with the best results for the BIS2 equation. The boxplots of change shows the difference between eGFR and mGFR and reconfirms these results for all seven eGFR equations (Supplement Fig. 1).

### Performance of BTP based estimating equations and comparison with established equations

Table 3 shows that for the BTP-based equations, bias and accuracy was best for the Inker_(BTP)_ equation and worst for the White_(BTP/Crea)_ equation. When comparing the new BTP-based equations with the established creatinine/cystatin C-based CKD-EPI_(Crea/CysC)_, BIS2 and FAS equations it was surprising that the Inker_(BTP)_ equation showed a better performance of bias (−2 versus 7 ml/min/1.73 m^2^) and accuracy (P10 of 45% versus 40% and P30 of 91% versus 88%) than the CKD-EPI_(Crea/CysC)_ equation. All in all the BIS2 showed the best performance, which is not surprising since it was developed from the BIS dataset, and the FAS was relatively equal to Inker_(BTP)_ and superior to CKD-EPI_(Crea/CysC)_. Both Pöge equations did not outperform the established creatinine/cystatin C-based equations.

**Table 3:** Bias, Precision, and Accuracy for eGFR Equations containing BTP in Individuals aged 70 years and above.

Equation	Mean Bias (ml/min/1.73 m^2^)	SD of Differences (ml/min/1.73 m^2^)	P10 (%)	P30 (%)
Pöge_(BTP)_	3.50	11.5	45.2	85.2
Pöge_(BTP/Crea)_	2.38	10.4	44.9	87.8
White_(BTP/Crea)_	25.88	13.0	3.4	25.6
Inker_(BTP)_	−2.00	10.9	44.9	90.5
BIS2*	−0.15	7.7	60.1	96.6
CKD-Epi_(Crea/Cys)_	6.95	8.9	39.6	88.2
FAS_(Crea/Cys)_	−0.93	11.9	32.2	91.2

Detailed description of GFR estimating equations can be found in the material section. BIS = Berlin Initiative Study, CKD-EPI = Chronic Kidney Disease, FAS = Full age spectrum. Bias was defined as difference between eGFR and mGFR for each equation. P10 and P30 refer to percentage differences [(eGFR − mGFR)/mGFR × 100]. *The results of the BIS2 differ slightly from former publications^3^ where the validation of the equation within the BIS data set was performed in only half of the iohexol population. For comparison reasons the current validation for BIS2 was done in the entire BIS iohexol population (n = 566) including the development sample leading to a slightly more favorable result.

### Comparison of BTP-based and creatinine/cystatin C-based equations

**Figure 3:**
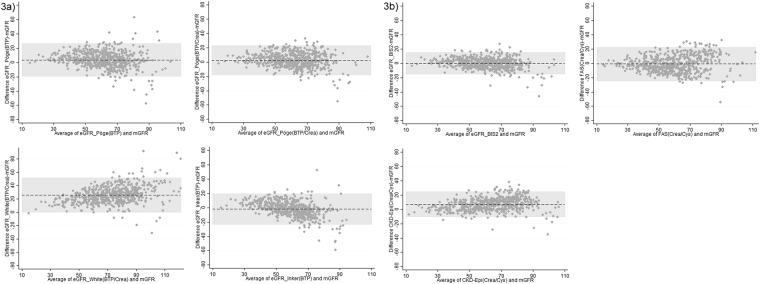
(**a**) Bland and Altman of BTP-based eGFR-equations versus mGFR. (**b**) Bland and Altman of creatinine-/cystatin C-based eGFR-equations versus mGFR. (**a**) and (**b**) Bland and Altman plots of BTP-based and combined creatinine/cystatin C-based eGFR-equations versus mGFR (n = 566). The bias is represented by the dashed middle line. The horizontal grey bar represents the area between the upper and lower limits of the interval of agreement. mGFR = measured glomerular filtration rate; eGFR = estimated glomerular filtration rate; BIS = Berlin Initiative Study; CKD-Epi = Chronic Kidney Disease Epidemiology Collaboration, FAS = Full Age Spectrum. For details about the GFR estimating equations please refer to the material section.

Figure 3a illustrates the performance of the four BTP-based GFR equations versus mGFR whereas Fig. 3b illustrates the three combined creatinine/cystatin C-based equations versus mGFR using Bland-Altman analysis. Also, both figures show that all GFR equations perform inferior at higher levels of GFR. Finally, exact McNemar significance probability of respective eGFR equations using P30 criterion can be found in Supplement Table 3a and b.

Subgroup analysis in individuals with arterial hypertension, diabetes mellitus, low or high body mass index demonstrated a slight additional benefit of including BTP into the prediction model for patients with arterial hypertension and diabetes as well as lean individuals (Supplement Table 2a,b).

## Discussion

Improving the accuracy of glomerular filtration rate (GFR) estimating equations has been a long-standing aim in order to optimize clinical decision making with regard to diagnosing chronic kidney disease (CKD), medication dose-adjustment, contrast media application, initiating renal replacement therapy, evaluation of potential organ donors as well as prognostication of CKD-associated CV risk - all scenarios very relevant in the elderly.

In the present study we investigated ß-trace protein (BTP), a relatively novel filtration marker for estimating kidney function, for the first time exclusively in older adults. This was motivated by the fact that introduction of cystatin C into current GFR estimating equations a couple of years ago has been shown to increase accuracy and precision especially in older adults^2–4,17^ most probably due to its independence of height, age, gender and muscle mass. While the appropriate renal biomarker in old age has yet to be established, this raised the question whether the addition of BTP could further optimize GFR assessment in elder patients to improve patient management in geriatric nephrology.

Our analysis was performed in 566 individuals aged 70 years and above with iohexol plasma clearance measurements. We demonstrated for the first time that when BTP was included into measured GFR (mGFR) predicting regression models, neither the single biomarker with or without age and gender was superior to creatinine or cystatin C, nor did BTP in combination with creatinine and/or cystatin C increase the regression model’s fit significantly.

When comparing all BTP-containing GFR equations to mGFR the Inker_(BTP)_^18^ equation showed the best performance. No additional benefit was found when evaluating the performance of BTP-based GFR estimating equations^15,16,18^ in our population of older adults compared to BIS2 and FAS_(Crea/Cys)_ equations^2–4^. Therefore, using the two “established” standardized biomarkers creatinine and cystatin C for estimating GFR in individuals above the age of 70 is still superior to the currently available BTP-based equations. When comparing the Inker_(BTP)_ to the creatinine/cystatin C-based CKD-EPI_(Crea/Cys)_^2^ equation we found that the Inker_(BTP)_ equation showed a substantially smaller bias and better precision. This was very surprising to us since in our mGFR predicting regression models we found that the combination of creatinine and cystatin C was clearly superior to BTP as a single biomarker in older adults. Therefore, we would have expected the established CKD-EPI_(Crea/Cys)_ equation to outperform the Inker_(BTP)_ equation. One possible explanation why this was not the case might be that the development population of both equations were not the same: amongst others, the Inker_(BTP)_ study population included individuals with a higher mean age and reported a diabetes prevalence of 21%, both of which corresponds more closely to the characteristics of our BIS population compared to the CKD-EPI study population. Also, one prominent non-GFR determinant in old age certainly is inflammation by which BTP is known to be less influenced compared to cystatin C^11^. This could be another possible explanation why the Inker_(BTP)_ equation performed better than the combined creatinine/cystatin C- based equation.

Interestingly, in contrast to former analyses that found no independent association between age and BTP^19^, our data showed that mean BTP concentration increases by 14% between the age of 70 and the age of 90 years and above, leading to a higher prevalence of GFR <60 ml/min/1.73 m^2^ in the higher age strata. The same phenomenon has been shown for creatinine and cystatin C^20^, and has been interpreted as a sign of physiological kidney senesence^21^. Cystatin C is said to be more sensitive to earlier GFR decrease^22^ in contrast to creatinine, which detects changes in kidney function not before a decrease of at least 50%. Priem *et al*. as well as Filler *et al*. have found the same advantage for BTP with regard to the “creatinine-blind” range^23,24^; in our population of elderly we did not find such a correlation (data not shown).

We further investigated BTP for assessing kidney function in hypertensive, diabetic, lean or obese elderly patients and found a slight additional benefit of including BTP into the prediction model for patients with arterial hypertension and diabetes as well as lean individuals compared to the combination of creatinine and cystatin C alone. All in all our data did not show that BTP, in addition to creatinine and cystatin C, contains significant additional information on kidney function enabling a better distinction between disease-related and age-related “physiological” change of kidney function. Besides, the accuracy of BTP measurement is lower compared to creatinine and cystatin C^25,26^ since a certified assay standardization has not yet been successfully established^27^, a fact that may also contribute to the rather disappointing results.

In 2016, Foster and colleagues have shown that BTP may contribute valuable risk prediction information beyond the current biomarkers creatinine and cystatin C^28^. As a purely cross-sectional analysis our focus exclusively lay on GFR prediction.

Several limitations of our study deserve mentioning. First, BTP has been shown to perform superior in combination with beta-2-microglobulin (B2M)^18^. Since in our population we did not measure B2M our observations are limited to BTP only or in combination with creatinine and/or cystatin C. Secondly, our study explicitly focused on older adults and BTP’s potential benefit in estimating GFR in this selected population. Even if we cannot simply expand our results to other populations they support former findings where BTP performed neither superior in children^13,29^ nor in adults^30,31^ when compared to creatinine and cystatin C. Also we cannot comment on the impact of race, since we investigated a purely Caucasian population. Thirdly, the BTP-based equation by Pöge^16^ and White^15^ were derived in and developed for adult kidney transplant recipients; these equations were expected to perform inferior in our elderly non-transplanted study population.

In summary, BTP alone or the addition of BTP does not outperform current biomarkers such as creatinine and cystatin C for GFR estimation in older adults. Especially the use of cystatin C renders the addition of BTP unnecessary. A slight improvement might be present in diabetic, hypertensive and lean individuals, whether this is clinically relevant remains to be proven. Further research is necessary to evaluate whether BTP is a useful renal marker in risk prediction for older adults and whether it outperforms creatinine and/or cystatin C in this respect.

## Methods

### Study participants

The study includes a subpopulation (n = 570) of the longitudinal population-based Berlin Initiative Study (BIS) cohort (n = 2,069). Participants, all living in Berlin, were recruited through one of the largest German statutory health care insurance company (AOK Nordost – Die Gesundheitskasse). Details on inclusion and exclusion criteria as well as study design and goals can be found elsewhere^32^. In the iohexol subpopulation of 570 out of 2,069 BIS baseline participants we performed iohexol plasma clearance for exact measurement of GFR. Out of these 570 participants we had 566 with measured serum ß-trace protein (BTP) as well as the two common renal biomarkers creatinine and cystatin C; in four patients BTP analysis was not performed due to lack of biobank material. The methodology used to measure GFR has been described elsewhere^3,33^. Briefly, an eight time point plasma iohexol measurement was performed over a five hour time period after i.v. injection. Iohexol was analyzed with high performance liquid chromatography (HPLC) method. Measured GFR (mGFR) was calculated with the area under the receiver-operating characteristic curve of plasma concentration over time. External quality control was provided by Equalis (Equalis AB, Uppsala, Sweden). Iohexol results were indexed for body surface area according to the DuBois and DuBois formula^34^. All procedures involving participants and data were in accordance with the revised Helsinki Declaration of 2000, concerning ethical principles for medical research involving human subjects. We confirm that the study protocols and patient information were approved by the local’s ethics committee of the Charité University.

### Laboratory measures

BTP measurement was done from frozen samples (−80 °C) at Labor Limbach Heidelberg, Germany, using the particle enhanced nephelometric (PENIA) N Latex® assay on the BN™ II System (Siemens Health Care Diagnostics, ex-Dade-Behring, Marburg, Germany) in 2015. The interassay coefficients of variation for serum BTP levels were 3.45% at concentrations of 1.76 mg/L.

Cystatin C measurements were performed from frozen samples at Charité laboratory, Labor Berlin, Germany, using the particle-enhanced nephelometric assay on the BN ProSpect nephelometer (Siemens Healthcare) in 2011. The interassay coefficient of variation for serum cystatin C levels were 1.5%, 3.5% and 2.4% at mean concentrations of 0.8, 2.3, 7.4 mg/L, respectively. The manufacturer’s reference interval for healthy subjects is 0.59–1.05 mg/L, after standardization of cysC according to ERM – DA 471/IFCC for BN Systems. All reagents and control material were provided by Siemens Health Care Diagnostics.

All creatinine samples were analyzed directly after the study visit at the Synlab MVZ Laboratory, Heidelberg, using the standardized isotope dilution mass spectrometry-traceable enzymatic method (CREA plus, Roche Diagnostics, Mannheim, Germany) on a Roche modular analyzer P-Module between 2010 and 2011. The interassay coefficient of variation for serum creatinine were 2.3% and 3.4% at mean concentrations of 0.99 mg/dL and 3.75 mg/dL, respectively.

### Equations that were used for validation of estimated GFR (eGFR)

The performance of the four currently published GFR estimating equations (including either serum BTP alone or in combination with creatinine) was calculated:

1. Inker_(BTP)_ equation^18^: *eGFR* = *55* × *BTP* ^−*0.695*^ × *0.998* ^*age*^ (×*0.899 if female*)
2. Pöge_(BTP)_ equation^16^: *eGFR* = *47.17* × *BTP* ^*−0.7933*^
3. Pöge_(BTP/Crea)_ equation^16^: *eGFR* = *974.31* × *BTP* ^−*0.2594*^ × *creatinine* ^−*0.6*^
4. White_(BTP/Crea)_ equation^15^: e*GFR* = *167.8* × *BTP* ^−*0.758*^ × *creatinine* ^*−0.204*^ (×*0.871 if female*).

For comparison purposes we also calculated eGFR values with the combined creatinine and cystatin C based (5) BIS2^3^, (6) CKD-EPI_(Crea/CysC)_^2^ and (7) FAS_(Crea/CysC)_^4^ equations.

### Statistical analysis

Descriptive analysis includes means, SDs, ranges or medians for continuous variables; absolute frequencies and percentages for categorical variables. We assessed Pearson and Spearman correlation coefficients of the log of each marker to the log of mGFR. In double logarithmic linear models prediction of mGFR (reference standard) by BTP only and in combination with serum creatinine and/or cystatin C were performed (including analyses adjusted for age, gender, and BMI). Subgroup analyses were performed in hypertensive (AHT), diabetic (DM), lean (BMI ≤ 23), and obese (BMI > 30) elderly individuals. We calculated the coefficient of determination (corrected R^2^) to quantify to which extent the inclusion of BTP would improve the fit of the model of mGFR. To investigate whether interaction between endogenous biomarkers and BMI was significant we defined three BMI categories (BMI ≤ 23, 23 < BMI < 30, BMI ≥ 30) and included them and their interaction terms with respective biomarkers into regression models adjusted for age and gender.

Additionally, confidence limits for R^2^ and Root Residual Mean Square Error (RMSE) were calculated via bootstrap resampling.

To examine how well the four current BTP-based GFR estimating equations performed in our population of older adults we assessed bias as the mean and median difference between eGFR and mGFR, with positive values indicating an overestimation of mGFR. Precision was assessed as interquartile range for the difference and SD of the bias, and accuracy as the percentage of estimates within 10% and 30% of mGFR (P10, P30). Mc Nemar test were applied to compare P30 values between equations.

Additionally, for comparison of estimated GFR versus measured GFR, we used Bland-Altman analysis including limits of agreement taking into account random measurement errors^35^.

The analysis was done using STATA (StataCorp. 2015. *Stata Statistical Software: Release 14*. College Station, TX: StataCorp LP).

### Data availability

The datasets generated during and/or analyzed during the current study are available from the corresponding author on reasonable request.

### Ethical approval and informed consent

We confirm that informed consent was obtained from all participants and/or their legal guardian/s.

## Electronic supplementary material

Supplementary Material
