# Author Correction: Beta Trace Protein does not outperform Creatinine and Cystatin C in estimating Glomerular Filtration Rate in Older Adults

**DOI:** 10.1038/s41598-019-43722-5

**Published:** 2019-05-09

**Authors:** Natalie Ebert, Camilla Koep, Kristin Schwarz, Peter Martus, Nina Mielke, Jan Bartel, Martin Kuhlmann, Jens Gaedeke, Markus Toelle, Markus van der Giet, Mirjam Schuchardt, Elke Schaeffner

**Affiliations:** 1 Institute of Public Health, Charité University Medicine, Berlin, Germany; 2 Institute of Clinical Epidemiology and Medical Biostatistics, Eberhard Karls University, Tübingen, Germany; 3 Limbach Laboratory, Heidelberg, Germany; 4 Department of Nephrology, Vivantes Klinikum im Friedrichshain, Berlin, Germany; 5 Division of Nephrology, Charité University Medicine, Campus Mitte, Berlin, Germany; 6 Division of Nephrology, Charité University Medicine Campus Benjamin Franklin, Berlin, Germany

Correction to: *Scientific Reports* 10.1038/s41598-017-12645-4, published online 04 October 2017.

This Article contains errors in the results of the Full Age Spectrum (FAS)_(crea/cys)_. For the original calculation of mean bias, SD of differences, P10 and P30 value as well as Bland Altman in Figure 3 the rescaling factor for Serum creatinine (Scr) was switched for females and males.

As a result, in the Results section, under subheading ‘Performance of BTP based estimating equations and comparison with established equations’,

“All in all the BIS2 showed the best performance, which is not surprising since it was developed from the BIS dataset, and the FAS was relatively equal to Inker_(BTP)_ and superior to CKD-EPI_(Crea/CysC)_.”

should read:

“All in all the BIS2 showed the best performance, which is not surprising since it was developed from the BIS dataset, and the FAS_(crea/cys)_ was relatively equal to BIS2 and superior to Inker_(BTP)_ and ECD-EPI_(crea/cysc)_.”

In addition, the correct Table 3, showing the correct details for the FAS_(Crea/Cys)_ row, is shown below as Table 1.

**Table 1:** Bias, Precision, and Accuracy for eGFR Equations containing BTP in Individuals aged 70 years and above.

Equation	Mean Bias (ml/min/1.73 m^2^)	SD of Differences (ml/min/1.73 m^2^)	P10 (%)	P30 (%)
Pöge_(BTP)_	3.50	11.5	45.2	85.2
Pöge_(BTP/Crea)_	2.38	10.4	44.9	87.8
White_(BTP/Crea)_	25.88	13.0	3.4	25.6
Inker_(BTP)_	−2.00	10.9	44.9	90.5
BIS2*	−0.15	7.7	60.1	96.6
CKD-Epi_(Crea/Cys)_	6.95	8.9	39.6	88.2
FAS_(Crea/Cys)_	−0.32	8.0	57.1	96.3

Detailed description of GFR estimating equations can be found in the material section. BIS = Berlin Initiative Study, CKD-EPI = Chronic Kidney Disease, FAS = Full age spectrum. Bias was defined as difference between eGFR and mGFR for each equation. P10 and P30 refer to percentage differences [(eGFR – mGFR)/mGFR × 100].

*The results of the BIS2 differ slightly from former publications^3^ where the validation of the equation within the BIS data set was performed in only half of the iohexol population. For comparison reasons the current validation for BIS2 was done in the entire BIS iohexol population (n = 566) including the development sample leading to a slightly more favorable result.

Finally, the correct Figure 3, containing the correct Bland-Altman plot for FAS_(Crea/Cys)_ in panel b, appears below as Figure 1.

**Figure 1:**
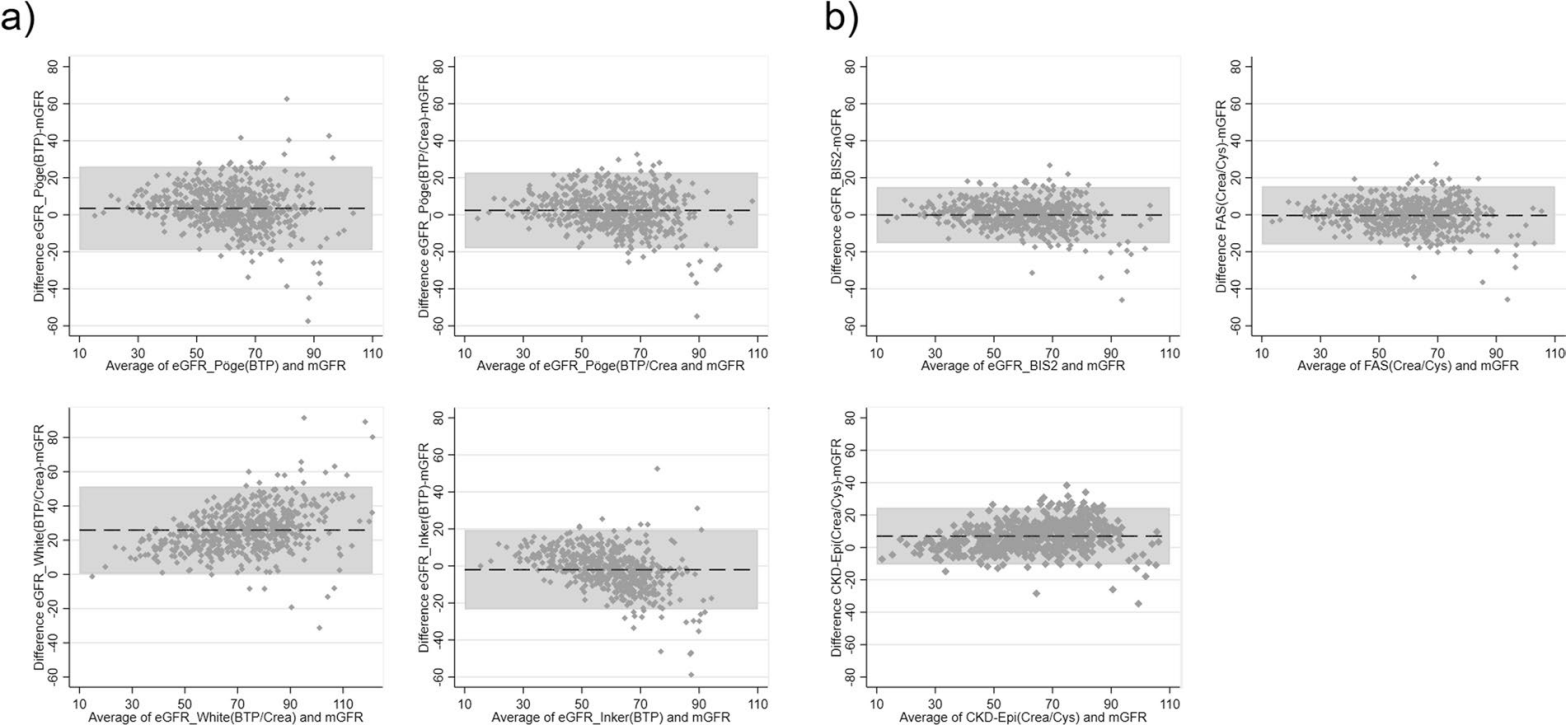
(**a**) Bland and Altman of BTP-based eGFR-equations versus mGFR. (**b**) Bland and Altman of creatinine-/cystatin C-based eGFR-equations versus mGFR. (**a**) and (**b**) Bland and Altman plots of BTP-based and combined creatinine/cystatin C-based eGFR-equations versus mGFR (n = 566). The bias is represented by the dashed middle line. The horizontal grey bar represents the area between the upper and lower limits of the interval of agreement. mGFR = measured glomerular filtration rate; eGFR = estimated glomerular filtration rate; BIS = Berlin Initiative Study; CKD-Epi = Chronic Kidney Disease Epidemiology Collaboration, FAS = Full Age Spectrum. For details about the GFR estimating equations please refer to the material section.

