# Supplementary material for: Beta Trace Protein does not outperform Creatinine and Cystatin C in estimating Glomerular Filtration Rate in Older Adults
Source: Sci Rep. 2017 Oct 4;7:12656. doi: 10.1038/s41598-017-12645-4 (PMC5627233; doi:10.1038/s41598-017-12645-4)
Supplement: Supplementary file 1 — Supplementary Material [file 41598_2017_12645_MOESM1_ESM.doc]

### Beta Trace Protein does not outperform Creatinine and Cystatin C in estimating Glomerular Filtration Rate in Older Adults

Natalie Ebert* MD, MPH^1^; Camilla Koep^1^; Kristin Schwarz MSc^1^; Peter Martus PhD^2^; Nina Mielke MSc, PhD^1^; Jan Bartel MD^3^; Martin Kuhlmann MD^4^; Jens Gaedeke MD^5^; Markus Toelle MD^6^; Markus van der Giet MD^6^; Mirjam Schuchardt PhD^6^ and Elke Schaeffner MD, MSc^1^.

^1^Charité University Medicine, Institute of Public Health, Berlin, Germany; ^2^Institute of Clinical Epidemiology and Medical Biostatistics, University Tübingen, Germany; ^3^Limbach Laboratory, Heidelberg, Germany; ^4^Department of Nephrology, Vivantes Klinikum im Friedrichshain, Berlin; ^5^Division of Nephrology, Charité University Medicine, Campus Mitte, Berlin; ^6^Division of Nephrology, Charité University Medicine Campus Benjamin Franklin, Berlin;

Corresponding author:

Natalie Ebert, MD, MPH

Charité University Hospital

Institute of Public Health - Campus Virchow

Seestr. 73, Haus 10

13347 Berlin, Germany

Phone: +49 (0)30 450 653209

Fax: +49 (0)30 450 7653209

Email: [natalie.ebert@charite.de](mailto:natalie.ebert@charite.de)

Running title: BTP as renal marker in elderly

Supplement Table 1 shows the performance of BTP alone and in combination with creatinine and cystatin C and compared to measured GFR (mGFR). Subgroup analysis in individuals with arterial hypertension, diabetes mellitus, low or high body mass index are presented in Supplement Table 2a and 2b. Exact McNemar test for assessing significance probability of respective estimated GFR (eGFR) equations using P30 criterion is shown in Supplement Table 3a (“1” was used for “within 30% accuracy” and “0” was used for “not within 30% accuracy”). The P30 values derived from the respective GFR equations are tested against each other and the ranking of GFR equations is done on the basis of descending P30 results, with the best result in the first row and the worst result in the last row.

Supplement Table 3b shows an example from Supplement Table 3a where the Pöge_(BTP)_ equation and mGFR is compared to the Pöge_(BTP/Crea)_ equation and mGFR. Pöge_(BTP)_ equation has a P30 value of 85.2%, Pöge_(BTP/Crea)_ equation performs superior with a P30 value of 87.8%. The difference though is not yet significant (p = 0.0864). We tested whether or not the difference N=41 and N=26 may have occurred randomly.

Finally, Supplement Figure 1 shows the box plot of change between eGFR and mGFR of the BTP-based and the combined creatinine/cystatin C-based GFR equations.

**Supplement Table 1:** Correlation Coefficients (Above grey diagonal: Pearson’s’ rho; below grey diagonal: Spearman’s rho) of log filtration markers (creatinine, cystatin C and BTP) against log mGFR and amongst themselves.

| Variable | mGFR | Creatinine | Cystatin C | BTP |
| --- | --- | --- | --- | --- |
| mGFR |  | -0.781 | -0.871 | -0.780 |
| Creatinine | -0.727 |  | 0.817 | 0.771 |
| Cystatin C | -0.835 | 0.761 |  | 0.832 |
| BTP | -0.732 | 0.716 | 0.787 |  |

mGFR = measured GFR, BTP = ß-trace protein

**Supplement Table 2a:** Double Logarithmic Linear Regression Models including the Combination of Creatinine and Cystatin C or Creatinine, Cystatin C and BTP for four Different Subgroups in Individuals aged 70 years and above (n=566).

| Subgroups | Regression model adjusted for age and gender | Corr. R^2^ | RMSE | p-value | | |
| --- | --- | --- | --- | --- | --- | --- |
|  |  |  |  | Crea | CysC | BTP |
| AHT^1^,  n= 435 | Crea + CysC  Crea + CysC + BTP | 0.819  0.829 | 0.126  0.122 | <0.001  <0.001 | <0.001  <0.001 | <0.001 |
| DM^2^,  n=136 | Crea + CysC  Crea + CysC + BTP | 0.849  0.861 | 0.124  0.119 | <0.001  <0.001 | <0.001  <0.001 | 0.001 |
| BMI^3^ ≤23,*  n= 53 | Crea + CysC  Crea + CysC + BTP | 0.885  0.896 | 0.113  0.107 | <0.001  <0.001 | <0.001  0.001 | 0.015 |
| BMI^3^ ≥30,  n= 158 | Crea + CysC  Crea + CysC + BTP | 0.830  0.833 | 0.123  0.122 | <0.001  <0.001 | <0.001  <0.001 | 0.065 |

Corrected coefficient of determination: r^2^, RMSE: Rout Square Mean Error;

^1^AHT: Arterial Hypertension defined as prescription of antihypertensive medication; ^2^DM: diabetes mellitus defined as either HbA1c >6.5%, and/or prescription of antidiabetic medication; ^3^BMI: body mass index. *Definition of “lean” individuals as BMI ≤23 is based on the fact, that the lowest percentile of BMI within the study population was defined as “lean”. BMI between 23 and 30 was defined as “normal” and BMI ≥30 as obese.

**Supplement Table 2b:** Standardized and Non-standardized Double Logarithmic Regression Models Adjusted for Age and Gender for BIS Subgroups with and without Diabetes and Hypertension and for lean (BMI ≤23), obese (BMI ≥ 30) and normal weight Patients (BMI 23 - 30).

| Subgroups | Regression model adjusted for age and gender | Standardized Coefficients | Non-standardized Coefficients | Standard Error |
| --- | --- | --- | --- | --- |
| AHT^1^, n= 435 | Crea +  CysC | -0.407  -0.530 | -0.414  -0.592 | 0.040  0.042 |
|  | Crea +  CysC +  BTP | -0.365  -0.421  -0.184 | -0.371  -0.471  -0.176 | 0.040  0.048  0.035 |
| no AHT^1^, n=131 | Crea +  CysC | -0.396  -0.536 | -0.403  -0.599 | 0.080  0.087 |
|  | Crea +  CysC +  BTP | -0.343  -0.459  -0.137 | -0.349  -0.514  -0.130 | 0.086  0.101  0.079 |
| DM^2^, n=136 | Crea +  CysC | -0.286  -0.642 | -0.307  -0.723 | 0.073  0.075 |
|  | Crea +  CysC+  BTP | -0.290  -0.494  -0.187 | -0.312  -0.556  -0.179 | 0.070  0.086  0.050 |
| No DM^2^, n=430 | Crea +  CysC | -0.423  -0.511 | -0.433  -0.575 | 0.041  0.044 |
|  | Crea +  CysC +  BTP | -0.370  -0.437  -0.142 | -0.378  -0.492  -0.138 | 0.044  0.050  0.041 |
| BMI^3^ ≤23*,  n= 53 | Crea +  CysC | -0.531  -0.554 | -0.574  -0.622 | 0.104  0.099 |
|  | Crea +  CysC +  BTP | -0.487  -0.393  -0.235 | -0.526  -0.441  -0.237 | 0.100  0.119  0.094 |
| 23<BMI^3^<30  n=355 | Crea +  CysC | -0.430  -0.494 | -0.448  -0.567 | 0.046  0.050 |
|  | Crea +  CysC +  BTP | -0.377  -0.391  -0.173 | -0.393  -0.449  -0.169 | 0.047  0.058  0.045 |
| BMI^3^ ≥30.  n= 158 | Crea +  CysC | -0.283  -0.652 | -0.282  -0.689 | 0.067  0.067 |
|  | Crea +  CysC +  BTP | -0.256  -0.599  -0.099 | -0.255  -0.633  -0.092 | 0.068  0.073  0.050 |

AHT: Arterial Hypertension defined as prescription of antihypertensive medication; DM: diabetes mellitus defined as either HbA1c >6.5%, and/or prescription of antidiabetic medication; BMI: body mass index. *Definition of “lean” individuals as BMI ≤23 is based on the fact, that the lowest percentile of BMI within the study population was defined as “lean”. BMI between 23 and 30 was defined as “normal” and BMI ≥30 as obese.

**Supplement Table 3a.** Exact McNemar significance probability of respective eGFR equations using P30 criterion (yes, within 30% accuracy = 1/ no, not within 30% accuracy =0).The rows are arranged with descending P30 values, with the first row showing the best results and the last one the worst result.*

| eGFR equation  (P30 value) | BIS2 | FAS_(Crea/CysC)_ | Inker_(BTP)_ | CKD-EPI_(Crea/CysC)_ | Pöge_(BTP/Crea)_ | Pöge_(BTP)_ | White_(BTP/Crea)_ |
| --- | --- | --- | --- | --- | --- | --- | --- |
| BIS2 (96.6%) |  |  |  |  |  |  |  |
| FAS_(Crea/CysC)_ (91.2%) | 0.00 |  |  |  |  |  |  |
| Inker_(BTP)_ (90.5%) | 0.00 | 0.7310 |  |  |  |  |  |
| CKD-EPI_(Crea/CysC)_ (88.2%) | 0.00 | 0.0363 | 0.2229 |  |  |  |  |
| Pöge_(BTP/Crea)_ (87.8%) | 0.00 | 0.0145 | 0.1147 | 0.9036 |  |  |  |
| Pöge_(BTP)_ (85.2%) | 0.00 | 0.0006 | 0.0000 | 0.1145 | 0.0864 |  |  |
| White_(BTP/Crea)_ (25.6%) | 0.00 | 0.00 | 0.00 | 0.00 | 0.00 | 0.00 |  |

BIS: Berlin Initiative Study, BTP: beta trace protein, CKD-EPI: Chronic Kidney Disease, FAS: Full age spectrum. P30 refers to percentage differences [(eGFR – mGFR) / mGFR x 100].

*See also Supp Table 3b.

**Supplement Table 3b:** This table shows an example from Supplement Table 3a where the Pöge_(BTP)_ equation and mGFR is compared to the Pöge_(BTP/Crea)_ equation and mGFR.

|  |  | Pöge_(BTP)_ equation vs. mGFR | | |
| --- | --- | --- | --- | --- |
|  |  | Diff ≤ 30% | Diff > 30% | Total |
| Pöge_(BTP/Crea)_ equation vs. mGFR | Diff ≤ 30% | N=456 | N=41 | 497 |
|  | Diff > 30% | N=26 | N=43 | 69 |
|  | Total | 482 | 84 | 566 |

**Supplement Figure 1.** Boxplots of Change between eGFR and mGFR of the BTP-based and the combined creatinine/cystatinC-based GFR equations


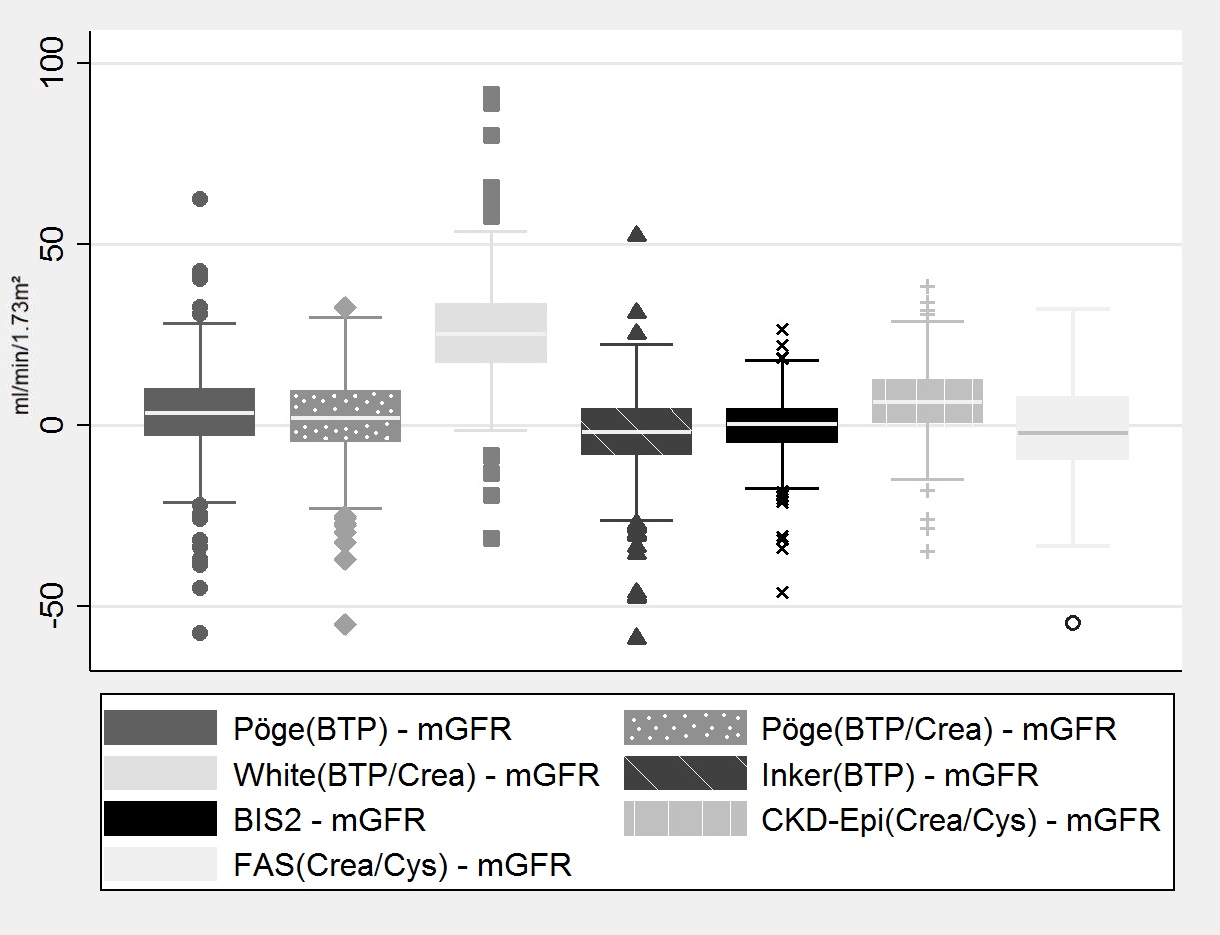


**Legend Supplement Figure 1:** Boxes indicate medians (line inside box), quartiles (upper and lower margins of box). Antennae are defined by the rule upper-lower box margin ± 1.5 x interquartile range. Circles, squares and triangles indicate outliers. For estimating equations, refer to material section. mGFR = measured glomerular filtration rate; BIS = Berlin Initiative Study; CKD-Epi = Chronic Kidney Disease Epidemiology Collaboration, FAS = Full Age Spectrum.
